# Dynamic Causal Modelling of epileptic seizure propagation pathways: A combined EEG–fMRI study

**DOI:** 10.1016/j.neuroimage.2012.05.053

**Published:** 2012-09

**Authors:** Teresa Murta, Alberto Leal, Marta I. Garrido, Patrícia Figueiredo

**Affiliations:** a Department of Bioengineering, Instituto Superior Técnico, Technical University of Lisbon, Lisbon, Portugal; b Institute for Systems and Robotics, Lisbon, Portugal; c Centro de Investigação e Intervenção Social, Lisbon, Portugal; d Department of Neurophysiology, Centro Hospitalar Psiquiátrico de Lisboa, Lisbon, Portugal; e Wellcome Centre for Neuroimaging, UCL, UK

**Keywords:** Epilepsy, Seizure, EEG–fMRI, DCM, Effective connectivity, Hypothalamic hamartoma

## Abstract

Simultaneous EEG–fMRI offers the possibility of non-invasively studying the spatiotemporal dynamics of epileptic activity propagation from the focus towards an extended brain network, through the identification of the haemodynamic correlates of ictal electrical discharges. In epilepsy associated with hypothalamic hamartomas (HH), seizures are known to originate in the HH but different propagation pathways have been proposed. Here, Dynamic Causal Modelling (DCM) was employed to estimate the seizure propagation pathway from fMRI data recorded in a HH patient, by testing a set of clinically plausible network connectivity models of discharge propagation. The model consistent with early propagation from the HH to the temporal–occipital lobe followed by the frontal lobe was selected as the most likely model to explain the data. Our results demonstrate the applicability of DCM to investigate patient-specific effective connectivity in epileptic networks identified with EEG–fMRI. In this way, it is possible to study the propagation pathway of seizure activity, which has potentially great impact in the decision of the surgical approach for epilepsy treatment.

## Highlights

► Seizure EEG–fMRI data of a hypothalamic hamartoma epileptic patient was analysed. ► Two competing hypotheses for seizure propagation pathways were tested. ► DCM was used to estimate all plausible connectivity models of seizure propagation. ► Bayesian model selection yielded a model consistent with previous evidence. ► This study provides the first application of DCM to EEG–fMRI data of human seizures.

## Introduction

Hypothalamic hamartomas (HH) are benign brain tumours located near the hypothalamus. Epileptic patients with HHs often experience gelastic or dacrystic seizures, which typically involve sudden bursts of energy in the form of laughing or weeping, not accompanied by the usual emotional sense of amusement or sadness, respectively (Berkovic et al., 1988). Previous studies have pointed towards HHs as the source of pathological activity (DiFazio and Davis, 2000; Fukuda et al., 1999; Kahane et al., 2003; Kuzniecky et al., 1997; Munari et al., 1995; Palmini et al., 2005). The complete control of seizures and improvement on behavioural disturbances can be achieved by resecting the HH, which is a reasonable choice compared to the relatively severe evolution of medically-treated HH associated epilepsy (Berkovic et al., 1988; Tassinari et al., 1997). In an unsuccessful HH resection, the interruption of seizure propagation through disconnection of the underlying pathways is an alternative surgical approach (Fohlen et al., 2003). However, the exact pathway is not known in general and it must be investigated in each patient.

Two alternative seizure propagation pathways have previously been described in HH patients: through the left fornix to the temporal lobe (Leal et al., 2003), or through the mammillo–thalamo–cingulate pathway to the frontal lobe (Kahane et al., 2003). The existence of these two pathways was proposed in one study, based on the observation of a consistent clinical and neurophysiologic pattern of either temporal or frontal lobe cortical secondary involvement, depending on whether the HH is connected to the mammillary bodies (temporal lobe cases) or to the medial hypothalamus (frontal lobe cases) (Leal et al., 2003). Another study reporting depth electrode recordings found an early propagation from the HH to the cingulate gyrus, probably mediated by the mammillo–thalamo–cingulate pathway (Kahane et al., 2003). These results would therefore be consistent with a frontal lobe pattern and associated propagation pathway. In contrast, in the study of a patient with a giant HH, an event-related synchronization/desynchronization (ERS/ERD) analysis of the electroencephalogram (EEG) suggested propagation from the HH directly to the left hippocampus and occipital lobe, and only later to the left cingulate gyrus and dorsal–lateral frontal lobe (Leal et al., 2009). This case study would therefore be consistent with a temporal lobe pattern and associated propagation pathway.

Simultaneous EEG correlated functional magnetic resonance imaging (EEG–fMRI) recordings have been successfully used to localise epileptic brain networks, through the identification of the blood oxygenation level dependent (BOLD) correlates of interictal (between seizures) and ictal (during seizures) electrical discharges (Donaire et al., 2009; Gotman et al., 2006; Salek-Haddadi et al., 2002). Characterisation of the BOLD signal spatial–temporal dynamics during seizures can be important for the identification of the epileptic focus and the direction of information flow within the network of functionally connected brain areas (Donaire et al., 2009; Szaflarski et al., 2010; Tyvaert et al., 2009). Dynamic Causal Modelling (DCM) is a biophysically informed framework suitable to study the influence that a neural system exerts over another (Friston et al., 1998). In the formulation of DCM for fMRI (Friston et al., 2003), the measured brain responses are integrated into a generative model that incorporates a dynamic model of interacting cortical regions, and a forward model of how the neuronal activity is transformed into the measured haemodynamic response. Non-biophysically inspired methodologies based on General Linear Modelling (GLM) have also been adopted to study seizure dynamics (Donaire et al., 2009; Szaflarski et al., 2010; Tyvaert et al., 2009).

In an outstanding study, David et al. (2008) employed DCM to study the propagation of excitation in a genetic rat model of absence epilepsy from EEG-correlated fMRI data, and compared the results with direct measures derived from subsequent intracerebral EEG recordings in regions strongly activated in fMRI. DCM was able to correctly predict the neural driver of generalized spike-and-wave discharges, despite considerable differences between haemodynamic delays among brain regions. So far, this work is the most direct experimental assessment of the validity of DCM for inferring network structure from fMRI data. Only two studies have previously employed DCM to study the dynamics of epileptic activity based on simultaneous EEG–fMRI recordings of human patients (Hamandi et al., 2008; Vaudano et al., 2009). In both studies, interictal activity was recorded and DCM was employed to identify the epileptic focus, through the comparison of competing connectivity models with different neural drivers. To the best of our knowledge, DCM has not been previously used to study seizure propagation in humans.

In this paper, we aimed to investigate the seizure propagation pathway in a medication-refractory epileptic patient with a giant HH using connectivity measures obtained from EEG-correlated fMRI data. DCM was applied and two competing hypotheses of seizure propagation were compared in terms of their capability to explain the data. A General Linear Model (GLM) methodology based on analysing the data at different time lags relative to the seizure onset was also employed.

## Methods

### Patient

#### General clinical characterisation

Our study focused on a patient with a giant HH undergoing pre-surgical evaluation in the scope of the Epilepsy Surgery Program of Hospital Egas Moniz, Lisbon (see Leal et al., 2009 for a related case report). The patient was a 2-year old boy whose first seizure occurred before the first year of life. His seizure semiology consists in motor arrest, rhythmic eyelid movements, fearful face, and occasional laughter. Seizures last less than 20 s and have a frequency of more than 50 daily. Background desynchronization, rhythmic spikes in left occipital lobe and later build up of rhythmic spikes in the left frontal lobe are characteristic of his ictal EEG recordings. Normal background with focal slow activity associated with abundant spikes over left occipital lobe is characteristic of his interictal EEG recordings. The patient was submitted to a two-stage HH disconnection. One year after the surgery, the frequency of seizures was reduced to 1–3 daily.

#### Seizure propagation pathway hypotheses

We assumed that the epileptic focus was known to be the HH (a well established finding in the HH literature and also in this case) and aimed to test the two previously proposed seizure propagation hypotheses: (I) from HH to the temporal–occipital, posterior region (PR) through the left fornix and from here to the frontal, anterior region (AR) through the left cingulate fasciculus (*HH* to *PR* to *AR*); and (II) from HH to the frontal, anterior region (AR) through the mammillo–thalamo–cingulate pathway and from here to the temporal–occipital, posterior region (PR) through the cingulate gyrus (*HH to AR to PR*).

### EEG–fMRI acquisition

The patient underwent a 1-hour simultaneous EEG–fMRI recording on a 1.5T GE Cvi/NVi MRI scanner. Six functional BOLD sequences were acquired using echo planar imaging (EPI) (TE/TR = 50/2275 ms, voxel size 3.75 × 3.75 × 5 mm^3^), each comprising 154 brain volumes (approximately 6 min), 150 of which were analysed after removal of the first 4. A structural T_1_-weighted image was also acquired using a 3D SPGR sequence for subsequent image registration.

The EEG was recorded using a 37-channel system (Maglink, Neuroscan, Charlotte, USA), with a sampling rate of 1 kHz and processed using a low-pass filter set at 70 Hz. Light anaesthesia using 1% sevoflurane (Abbott Laboratories, Abbott Park, IL, USA) was applied through a mask, in accordance to the MRI centre protocol for small children and uncooperative patients. A 5-minutes EEG was also recorded outside the scanner and before anaesthesia.

**Fig. 1:**
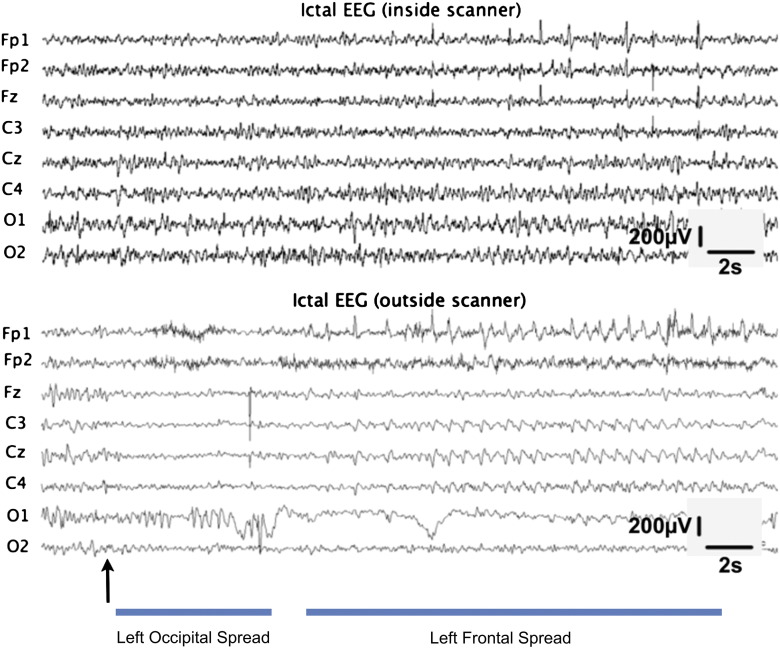
Typical ictal EEG recordings of the patient, obtained inside (above) and outside (below) the scanner. The ictal event is associated with an early central desynchronization, followed by rhythmic left occipital spikes and the build-up of left frontal spikes at later stages. The two traces are aligned with each other and the arrow indicates the seizure onset.

The EEG recordings inside the scanner were processed using the Scan 4.3 software (Maglink, Neuroscan, Charllote, USA) for removal of bad channels and artefact correction. These recordings were compared with the ones outside the scanner (Fig.1) for cross-validation, and the ictal events were then identified by visual inspection of the artefact-corrected recordings. The EEG recordings of the seizure inside the scanner are not exactly identical to the ones recorded outside the scanner. However, we have evidence that all of the patient's seizures are similar and therefore the discrepancies likely explained by contamination with scanner artefact. Two seizures occurred (6.8 to 9.1 s durations) in BOLD *Sequence 1* and five seizures occurred (9.1 to 18.2 s durations) in BOLD *Sequence 2*.

### Seizure-related haemodynamic changes

Pre-processing of BOLD data was performed using FSL (www.fmrib.ox.ac.uk/fsl/) and included: motion correction; slice timing correction; non-brain removal; spatial smoothing (8 mm FWHM Gaussian kernel); and high-pass temporal filtering (100 s cut-off).

**Fig. 2:**
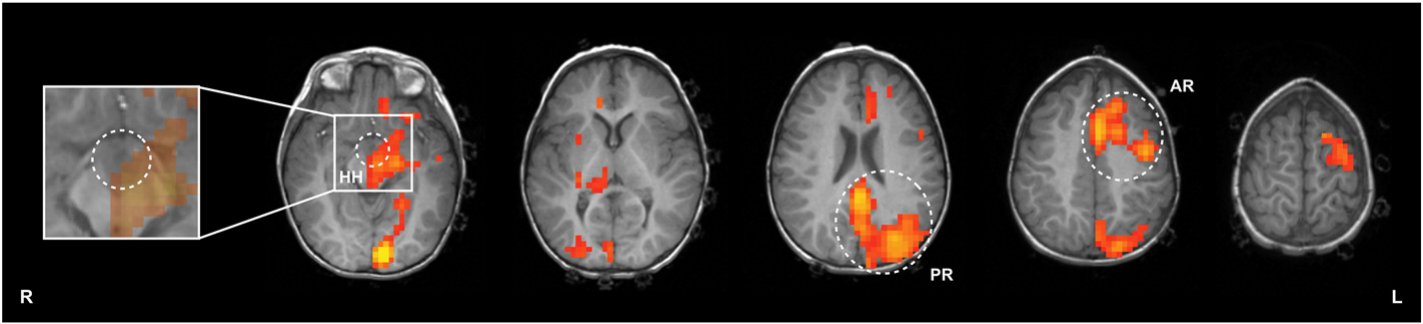
High-level t-map of seizure-related positive BOLD signal changes (cluster of 8 voxel minimum extent, voxel p < 0.001, uncorrected) overlaid on the T_1_-weighted structural of the patient. The location of three ROI's (HH, PR and AR) is highlighted with dashed circles. A zoom in is included to better demonstrate the overlap of the t-map with the HH.

The GLM analysis was performed using SPM8 (www.fil.ion.ucl.ac.uk/spm/software/spm8/). The square waveforms corresponding to the periods of ictal activity were convolved with a single-gamma haemodynamic response function (HRF) to build the regressor describing ictal-related BOLD signal changes. The corresponding temporal derivative was also considered as a regressor and motion parameters were further added as regressors of no interest. The GLM parameters were estimated using classical restricted maximum likelihood (ReML) estimation (Friston et al., 2002). A t-contrast for the ictal regressor was used to identify significant positive BOLD signal changes correlated with the ictal events. A fixed effects (FFX) high-level analysis was performed on the two data sequences. The resulting statistical parametric maps (SPMs) were thresholded at p < 0.001, uncorrected, and clusters with a minimum of 8 contiguous voxels were considered. The high-level SPMs are shown in Fig. 2.

### Definition of anatomical regions of interest (ROIs)

In order to test the competing hypotheses of seizure propagation, and based on the regions found to exhibit seizure-related BOLD signal increases, three anatomical regions of interest (ROIs) were defined, as highlighted in Fig. 2: the hypothalamic hamartoma (HH); an anterior region corresponding to the left frontal lobe (AR); and a posterior region including the left hippocampus, left occipital lobe, precuneus and posterior division of cingulate gyrus (PR).

The PR and AR anatomical masks were obtained in standard MNI space (Lancaster et al., 2007) using the atlases included in the FSL tool. The HH, a focal malformation, was visually identified on the high-resolution T_1_-weighted image and the associated anatomic mask was manually designed to specifically match its form. The *Sequence 1* images were registered to the *Sequence 2* images, and these were registered to the T_1_-weighted image, which was in turn registered to the MNI standard space. All registrations were performed with the FSL tool FLIRT (Jenkinson and Smith, 2001). Applying the transformation matrices, the anatomical masks were then registered into the functional imaging space.

### DCM analysis

**Fig. 3:**
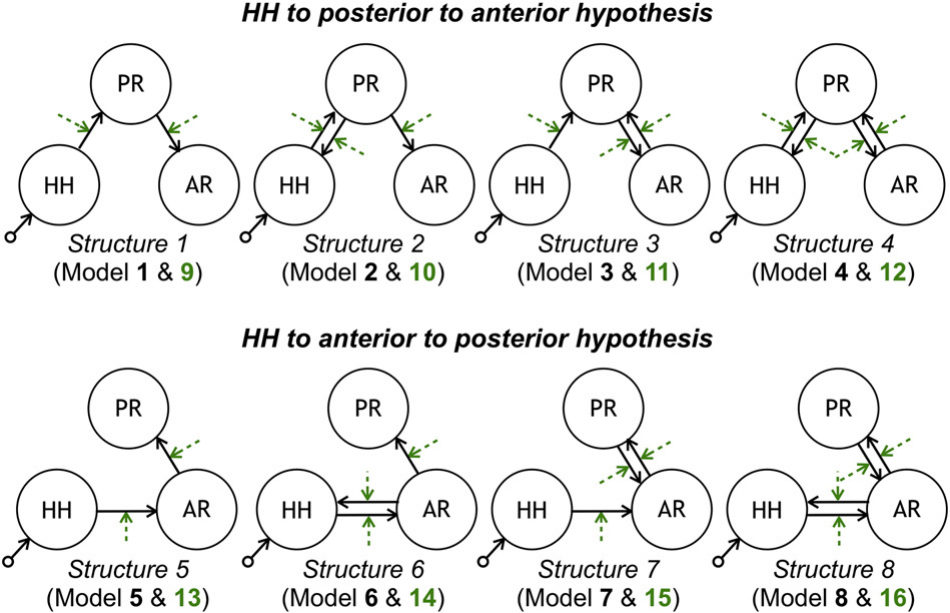
Model space tested with DCM. Each row contains eight models consistent with each propagation hypothesis. Each column corresponds to a different latent connectivity structure. For each latent connectivity structure, the linear model is presented with solid arrows and the bilinear model is presented with solid arrows (intrinsic connections) and dashed arrows (connections' modulation). Seizure activity is fed into the HH network node.

A space of sixteen connectivity models describing the two competing hypothesis for seizure propagation (I) and (II) was defined, as shown in Fig. 3.

The DCM analysis was performed using the DCM8 module included in SPM8. For this purpose, HH, AR, and PR functional ROIs were defined, for each sequence, based on the intersection of the respective SPMs of seizure-related BOLD increases, with the corresponding anatomical masks. For each ROI, a representative time-series was computed as the first eigenvector of all super-threshold voxel time-series within the ROI, in order to include the functionally relevant voxels.

In DCM for fMRI, the neuronal activity in each region, *z*, is described by the state equation:

(1) $$\dot{z}=Az+\Sigma u_{j}B^{j}z+Cu$$

where *u* is the external input to the region; matrix *A* is the latent connectivity, i.e., the intrinsic coupling in the absence of an extrinsic input; matrices *B*^*j*^ represent the modulation of connectivity by the external inputs *u*_*j*_, i.e., the change in coupling induced by the *j*^*th*^ input; and *C* embodies the influence of the extrinsic input to the region on its neuronal activity. In this study, a single driving region (the epileptic focus) was considered in all models, which means that the system's input *u* was exclusively fed to the HH node. The square waveforms corresponding to the periods of ictal activity were used as the system's input. Two possible directionalities were considered for each connection, unidirectional or bidirectional, which yielded a total of four connectivity structures consistent with each seizure propagation hypothesis. For each of these connectivity structures, two types of connectivity models were then considered: the linear models, which had only linear terms (*A* parameters); and the bilinear models, which had linear and bilinear terms (*A* and *B* parameters).

Firstly, inference on model structure was performed using Fixed Effects Bayesian Model Selection (FFX BMS) (Penny et al., 2004), in order to compare the individual models over the two BOLD sequences of interest (*Sequence 1* and *Sequence 2*). We used a FFX BMS over the two BOLD sequences because all seizures from the patient are comparable and therefore we expect that the model generating the data in each sequence should be the same. The model's Free Energy, F, a lower bound of the model's log-evidence, accounting for model complexity as well as data fit, was used to compare the likelihood of the different models to explain the data. Relative log-evidences, or differences in F, were converted into model/family posterior probabilities, p, indicating that the respective model/family has a probability p of being the best model/family explaining the data amongst all considered. Evidence was “strong” if p > 0.95, which stands for a difference in F greater than 3, and “positive” if 0.75 < p < 0.95, which stands for a difference of F between 1 and 3 (Penny et al., 2004, 2010).

Secondly, inference on the optimal model parameters was performed. The structure of the connectivity model was assumed to be the same for both sequences and a FFX analysis of the model parameter estimates was performed using Bayesian Parameters Averaging (BPA) (Acs and Greenlee, 2008; Garrido et al., 2007; Neumann and Lohmann, 2003). Additionally, a FFX family inference analysis (Penny et al., 2010) was performed by grouping the models according to: 1) propagation hypothesis (HH to PR to AR; HH to AR to PR); 2) latent connectivity scheme (each family included the pair of linear and bilinear models with the same scheme of latent connectivity); 3) connection directionality (both unidirectional; one uni- and one bi-directional; both bidirectional); and 4) model's linearity (linear; bilinear).

The choice of SPM significance threshold level is not straightforward since there is no universally accepted p value in the literature (the same applies to multiple testing control: FDR, FWE, etc.). On the other hand, the identification of the seizure onset by visual inspection of the EEG is subjective. In order to investigate the sensitivity of the DCM analysis to both factors, additional DCM analyses were performed by varying each of these parameters: three p values (0.05, 0.01, and 0.005) and nine seizure onset times (obtained by shifting the seizure regressor originally defined by the neurophysiologist by multiples of TR between − 4TR and + 4TR).

### Lagged GLM analysis

The Lagged GLM methodology consisted on analysing the data at different time lags relative to the seizure event (Szaflarski et al., 2010). A total of twenty-two GLMs were defined by shifting the reference seizure regressor, originally defined by the clinical neurophysiologist, by time lags ranging from − 11.4 to + 12.5 s in steps of 1.14 s (TR/2). Therefore, each GLM yielded the haemodynamic changes associated with a particular time lag relative to seizure onset. Following the methodology as originally proposed (Szaflarski et al., 2010), no further correction of voxel p values were considered to account for the 22 GLMs because no quantitative voxelwise comparisons between maps were being made. In order to observe the relative temporal evolution of haemodynamic changes in each ROI, the signal change associated with the regressor of interest was computed as a function of time lag and averaged within each anatomic ROI.

## Results

The SPM of seizure-related BOLD signal positive changes obtained with the conventional GLM analysis is first presented, followed by the results obtained by the DCM and Lagged GLM analyses.

### Seizure-related haemodynamic changes

The map of seizure-related BOLD signal increases obtained with a FFX conventional GLM high-level analysis is shown in Fig. 2. The movement was negligible in both sequences (absolute mean displacements of 0.05 mm). Significant BOLD increases were observed in the HH, precuneus, posterior division of cingulate gyrus, left hippocampus and contiguous left occipital lobe (corresponding to a posterior region, PR), and left frontal lobe (corresponding to an anterior region, AR). The location of seizure-related haemodynamic positive changes was consistent across the two data sequences. A more extensive thresholded SPM was obtained for *Sequence 2* (932 voxels), compared to *Sequence 1* (372 voxels), probably as a consequence of its greater number of ictal events, resulting in higher signal-to-noise ratio (SNR) and detection sensitivity for BOLD increases.

### DCM analysis

**Fig. 4:**
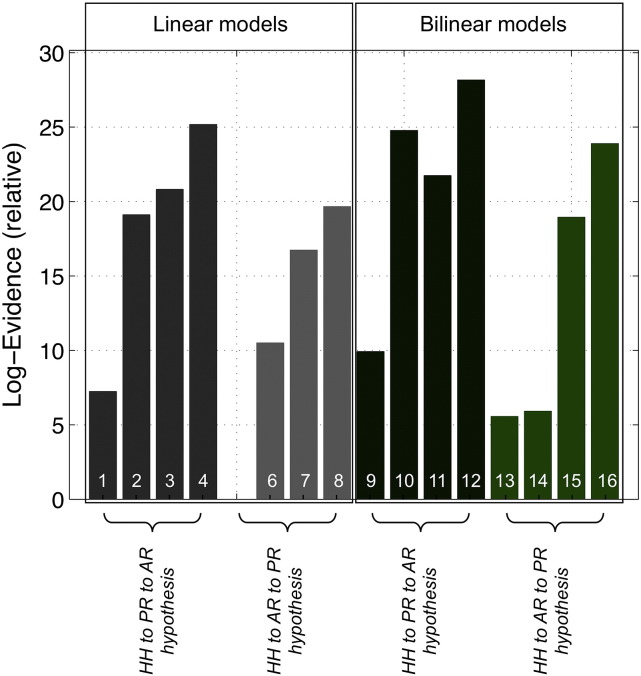
DCM Bayesian model selection results. Relative log-evidence for the sixteen models (Fig. 3) compared using FFX BMS. Models are grouped according to the propagation hypothesis, as indicated, and are separated into linear (left) and bilinear (right) models.

The FFX BMS results (models relative log-evidences) are presented in Fig. 4. The winning model is model 12, with a log-evidence difference relative to any one of the remaining models greater than 2.99. This indicates that model 12 is, with “positive” evidence, the best model explaining the data. The individual DCM results, obtained by performing BMS using each one of the two sequences separately, were also in agreement, although not conclusive (p = 0.33 for sequence 1 and p = 0.74 for sequence 2). The winning model is consistent with the seizure propagation hypothesis (I), which indicates that the epileptic activity propagates from the HH to the left hippocampus and contiguous left occipital lobe, to the precuneus and the posterior division of cingulate gyrus (comprising PR), reaching only afterwards the left frontal region (comprising AR). Moreover, the winning model has forward and backward connections between HH and PR and between PR and AR, and these are modulated by seizure activity through bilinear connectivity terms.

**Fig. 5:**
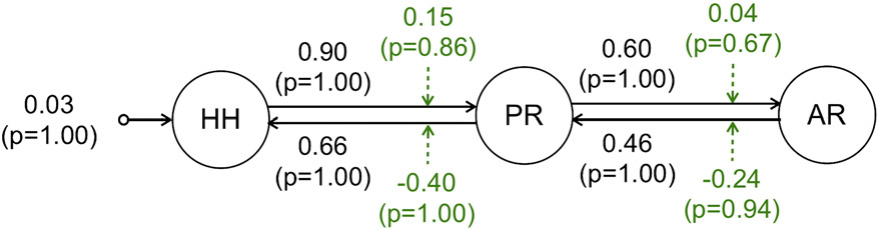
DCM Bayesian parameter averaging results. Averaged parameters obtained by FFX BPA for model 12, the best model according to FFX BMS. Averaged modulation parameters are indicated with dashed arrows and both intrinsic connectivity and the averaged direct input parameters are indicated with solid arrows.

The winning model FFX BPA results are shown in Fig. 5. With respect to the connectivity modulation by seizure activity, characterised by the bilinear *B* parameters, the values of the average parameters suggested that the strength of connections is enhanced in the directions HH to PR and PR to AR, and it is diminished in the opposite directions PR to HH and AR to PR. The posterior mean values of the estimated haemodynamic parameters, obtained for the winning model in each ROI and BOLD sequence, are presented in Table 1. No important differences can be found in the values of each parameter across ROIs or sequences.

**Fig. 6:**
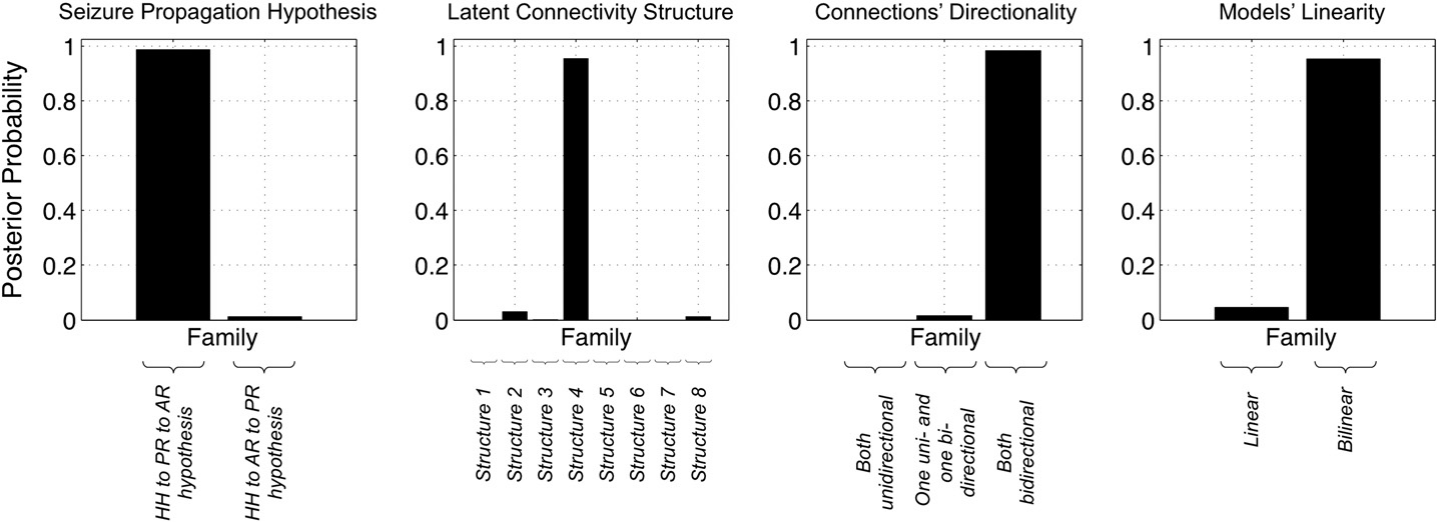
DCM FFX family inference results, according to: seizure propagation hypothesis; latent connectivity structure; connections directionality and model linearity.

FFX family inference results are presented in Fig. 6. In terms of the propagation hypothesis, the FFX family inference results support with “strong” evidence that models corresponding to the HH to PR to AR hypothesis (p = 0.99) are more likely than models corresponding to the HH to AR to PR hypothesis (p = 0.01). In terms of the latent connectivity structure, the results support, with “strong” evidence, that models 4 (linear) and 12 (bilinear), which have both forward and backward connections and correspond to propagation hypothesis (I), are more likely (p = 0.95) than models 8 (linear) and 16 (bilinear), which have both forward and backward connections and correspond to propagation hypothesis (II) (p = 0.01), as well as all of the remaining models. In terms of connection directionality, the family of models with both bidirectional connections was found to be more likely (p = 0.98) than the families with either only one or no bidirectional connections. Finally, in terms of model linearity, the results provided “strong” evidence for the family of bilinear models (p = 0.95) relative to its linear counterpart, suggesting that seizure activity modulates the strength of connections between network nodes.

The sensitivity study showed that the DCM results were consistent across SPM thresholding p values and seizure onset times, systematically yielding model 12 as the best model explaining the data. As expected, weaker evidence, and eventually inconclusive results or no seizure-related BOLD increases, were obtained as the onset was shifted away from the central onset time. Furthermore, we found that the HRF curves obtained with the haemodynamic parameters estimated for the winning model did not vary significantly across ROIs.

### Lagged GLM analysis

**Fig. 7:**
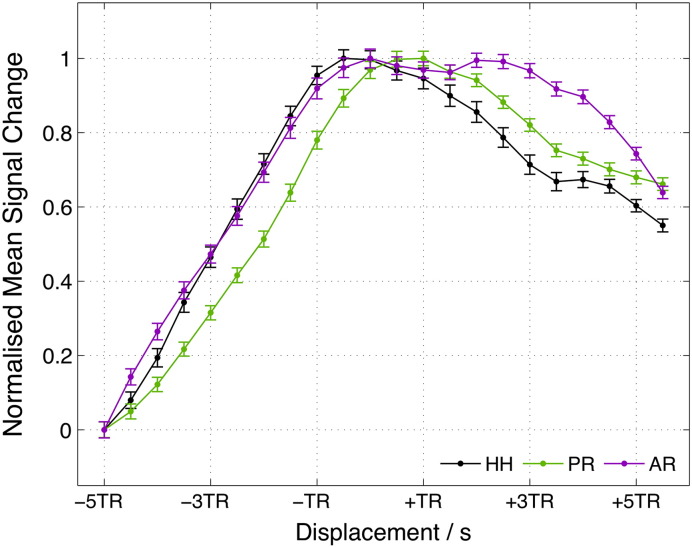
Lagged GLM analysis results. Normalised mean signal change values within each ROI as function of time lag (seizure onset displacement). Error bars represent standard error of the mean.

Lagged GLM results are presented in Fig. 7. The time lag at which the mean signal change reached its maximum was shorter for HH compared to PR, suggesting propagation of pathological activity from HH to PR. However, the peak of the mean signal change in AR was not clear, due to the presence of two local maxima, and hence propagation to AR could not be disentangled. Moreover, the AR and PR peaks are not distinguishable in time, since the maximum signal change in each ROI is not significantly different from its value at the position of the other ROI's peak (p > 0.3).

The effect of ROI size was investigated by reducing the size of the ROIs through the intersection of the thresholded SPMs (p < 0.001, uncorrected, 8 voxels extent) with the anatomical regions. We found that the lagged GLM results are also inconclusive using the smaller ROIs (results not shown).

## Discussion

Our work represents the first application of DCM to investigate seizure propagation pathways based on EEG-correlated fMRI recorded in humans. Two competing hypotheses for the causal chain leading to epileptic activity propagation in a patient with a giant HH were tested, based on clinically plausible scenarios previously described in the literature. The DCM results yielded propagation from the HH to a temporal–occipital, posterior region followed by a frontal, anterior region as the most likely model explaining the data.

Although the seizure focus may be the question of interest in most EEG-correlated fMRI studies of epileptic patients, this is not the case in HHs. In fact, the association between gelastic seizures and HHs is one of the strongest anatomical–clinical correlations in the field of human epilepsy. This focus location in the HH was also in agreement with the clinical history of our patient, as well as with the seizure reduction observed one year after the surgical two-stage hamartoma disconnection. The question of interest in the case of HHs is concerned with the pathway of seizure propagation from the HH to an extended brain network. Two alternative pathways have been shown in different patients, probably as a function of the exact location of the hamartoma within the hypothalamus (Kahane et al., 2003; Leal et al., 2003). The identification of the specific propagation model in individual patients would open surgical alternatives targeting the underlying propagation pathways, instead of the removal of the hamartoma in high-risk patients.

The dynamics of the epileptic activity propagation experienced by our patient was previously investigated through an ERS/ERD analysis of the EEG signal recorded during seizures outside the fMRI scanner (Leal et al., 2009). The involvement of the frontal lobe was always limited to the late phases of the EEG seizure event and was always preceded by left side occipital–temporal spike activity. It was then proposed that antidromic conduction in the fornix formation projecting to the posterior hippocampus could explain the focal slowing and abundant interictal spike activity over the left occipital and posterior temporal lobes. The left cingulate fasciculus would then conduct activity from posterior temporal to frontal lobe, overlapping the Papez circuit. These findings are therefore consistent with the results we obtained with the DCM analysis of EEG-correlated fMRI data regarding the pathway for seizure propagation, suggesting that the mammillo–hippocampus–cingulated pathway (hypothesis (I)) provides a better explanation of the data than the alternative mammillo–thalamo–cingulate pathway (hypothesis (II)).

By considering the pool of all plausible latent connectivity schemes within the three node network associated with seizure-related BOLD signal changes, our results indicated that both forward and backward connections played a role in seizure propagation. Besides having bidirectional connections, the winning model integrated bilinear terms describing the modulation of connectivity by seizure activity. Specifically, Bayesian parameter averaging showed an increase in the strength of forward connections and a decrease in the strength of backward connections during seizures. An intensification of the strength of forward connections could be related with seizure propagation through reduction in forward inhibition (Kuzniecky et al., 1997). It should be noted however that, in the DCM for fMRI formulation used here, the coupling parameters should be interpreted as a lumped measure of effective connectivity, and not as separate excitatory and inhibitory connections. This is in contrast with what happens in DCM for ERPs (M/EEG) (David et al., 2006), where excitatory and inhibitory connections are explicitly modelled, as well as in a recent development of DCM for fMRI (Marreiros et al., 2008).

In general, model misspecification is a potential problem in a DCM approach because this was designed to test a restricted space of connectivity models (Stephan et al., 2010). Bayesian model selection is strongly hypothesis-driven and the choice of the models to be tested should therefore be strictly based on the postulated hypotheses. Indeed, since no specified model is ever exactly correct (perfect fit to data), the purpose of model selection is to determine which model, from a set of plausible alternatives, best explains the data, i.e., represents the best balance between accuracy and complexity (Pitt and Myung, 2002). The choice of a set of plausible models is therefore critical, and it should be made so as to answer a specific research question. If an implausible model is included in the model space, it is possible that it is found to be the best at explaining the data; however, this result is not useful to answer the question at hand (Friston et al., 2011a). Importantly, all the models must have equal a priori probabilities (or otherwise known ones) in order to ensure BMS validity, since this is based on comparing the likelihood ratio under flat priors on model probabilities (i.e., $p(m_{1})=p(m_{2})$. The Bayes factor is defined as:

$$\frac{p(m_{1}|y)}{p(m_{2}|y)}=\frac{p(y|m_{1})p(m_{1})}{p(y|m_{2})p(m_{2})}$$

where *y* are the data and *m*_1, 2_ are the competing models 1 and 2. In our case, we wished to find out which one of the two previously proposed pathways was most likely to explain the seizure propagation in this patient. The two competing propagation hypotheses were therefore strongly physiologically sound and with the same a priori likelihood, and the specified three node network allowed us to test them. All the plausible models consistent with each of these two hypotheses were included in the tested model space.

The spatial definition of ROIs is also an important step. The connectivity network must be carefully defined based on a clear hypothesis. In our case, the ROIs were defined so as to fully partition the SPMs of seizure-related positive BOLD signal changes, according to the three pre-specified anatomical nodes. Although the identified brain network could be sub-divided in different ways (and eventually into different numbers of ROIs), and additional regions could have a profound impact on the winning model, we believe that the three ROIs considered here clearly allow us to test the two propagation hypotheses.

The generalisability of the proposed DCM approach for the study of seizure propagation strongly depends on the size of the brain network of interest and hence the feasibility of testing all possible connectivity models. This problem is common to applications of DCM in general and recent developments have been proposed to address it (Friston et al., 2011b). Moreover, we believe that such a DCM methodology should be well suited whenever well-defined a priori hypotheses regarding seizure propagation are available, which may be used to restrict the number of competing models to a tractable set.

One related concern with DCM approaches is that, if the model space does not include the true model, then the results might be biased towards the most complex models. Although the winning model in our case was in fact the most complex model, we have tested all possible, biologically motivated seizure propagation models within the network of seizure-related haemodynamic changes, and model complexity was taken into account in the Bayesian model selection methodology employed here. In any case, regardless of model complexity, the family of models consistent with propagation hypothesis (I) is more likely than the corresponding family consistent with propagation hypothesis (II). Also, the winning model, model 12, is more likely than the model of equal complexity corresponding to the opposite propagation hypothesis, model 16. This constitutes our main result.

One limitation of our study is that the implementation of DCM used presumes ictal activity as an extrinsic input, which is obviously not true for such an endogenous type of activity. The recent developments in stochastic DCM (Friston et al., 2011b; Li et al., 2011) may provide more suitable approaches for modelling spontaneous epileptic activity. Nevertheless, the system's input can be conceived as a time marker of an initial event taking place within the focus and which perturbs the postulated network. Similarly to previous studies (David et al., 2008; Hamandi et al., 2008; Vaudano et al., 2009), we have therefore defined the system's input as a square waveform corresponding to the periods of ictal activity identified on the EEG (Kobayashi et al., 2006; Tyvaert et al., 2008). Despite being the most common approach, this single-block scalp EEG derived fMRI model has its limitations (Thornton et al., 2010). The seizure onset is not always accurately detected by scalp EEG and may be delayed (Binnie and Stefan, 1999) and a single-block almost certainly does not represent dynamic processes such as seizures (Niedermeyer, 1999). Due to the shape of the input function (blocks), our analysis focused on the dynamics at the onset/offset of ictal events and is not designed to account for the dynamics of seizure evolution, i.e., between onset and offset.

The Lagged-GLM results suggested that epileptic activity propagated from the hamartoma to the posterior region, but it was not possible to clearly identify the temporal relation between the BOLD increases in these regions and the frontal lobe. Our results add to previous reports using similar approaches for the identification of the dynamics of seizure propagation (Donaire et al., 2009; Szaflarski et al., 2010; Tyvaert et al., 2009). The Lagged-GLM methodology has the advantage of accounting for some uncertainty in the definition of the beginning/end of the ictal events, as well as for a potential non-causal relationship between the EEG and BOLD events (Grouiller et al., 2010). However, this methodology lacks a supporting, biophysically informed connectivity model and may be strongly biased by differences in the haemodynamics of different brain regions (see David et al., 2008). In our study, we verified that the HRF curves estimated within the DCM framework showed no significant differences across ROIs. Otherwise, the HRF could have been estimated in each ROI and deconvolved from the respective BOLD time-series, before inclusion in the GLM (David et al., 2008).

It should be noted that it is not expectable that the DCM and time-lagged analyses should necessarily agree. In fact, the two methodologies approach causality in two distinct ways. While time-lagged analyses are based on temporal precedence, in which causes precede their consequences, DCM relies on both temporal precedence and physical influence, in which changing causes changes their consequences (Valdes-Sosa et al., 2011). Moreover, it has recently been suggested that time-lag based methods may not be adequate for the study of effective connectivity in fMRI (Smith et al., 2011). Therefore, we do not believe that our lagged GLM analysis should be considered as a standard against which the DCM analysis is compared.

Other fMRI data studies have used different methods to model ictal events and study the propagation of epileptic activity. Tyvaert et al. (2009) employed a unique GLM embodying a set of successive gamma function regressors, in order to test the contribution of different brain regions at different periods of time during the seizure. Donaire et al. (2009) divided each seizure epoch into blocks shifted relative to each other and analysed the BOLD changes between two consecutive blocks. Bai et al. (2010) analysed mean fMRI time courses to find maximal signal changes relative to seizure onset across the brain, which allowed them to characterise a complex sequence of early and late fMRI changes not detectable by conventional analysis. LeVan et al. (2010) decomposed the fMRI data by independent component analysis (ICA) to circumvent the predefinition of an HRF and subsequently estimated the HRF peak time in each identified region, in order to distinguish early responses in the onset zone from later propagated activity.

One concern in our study arises from the fact that light anaesthesia with sevoflurane was applied during the simultaneous EEG–fMRI recording. This procedure was not expected to reduce the epileptic activity significantly (Komatsu et al., 1994). Indeed the interictal and ictal events identified on the EEG acquired under light anaesthesia were comparable, in morphology and topography, to identical events identified on the EEG acquired without anaesthesia. This observation indicates that the seizures recorded using EEG–fMRI corresponded to the patient's typical epileptic activity and not to any pharmacologically induced abnormal rhythms. As far as the BOLD effect is concerned, previous studies measuring cerebral blood flow (CBF) and EEG simultaneously in the rat brain found a significant effect of anaesthesia on the HRF (Martin et al., 2006; Masamoto et al., 2009). In our study, the regional HRFs were estimated within the DCM framework and therefore our results should not be affected by such effect. Moreover, Liu et al. (2011) found that spontaneous CBF/BOLD fluctuations under unconscious burst-suppression anaesthesia conditions originated mainly from underlying neural activity. In our study, no significant changes in the EEG base rhythms were observed due to the light anaesthesia, and therefore the measured BOLD effects are not expected to be affected by it. One other limitation of the present study was the small number of seizures recorded. In fact, both GLM and DCM analyses were more sensitive for the sequence with more seizures, indicating that the number of interesting events is important.

In conclusion, our study indicates that DCM may offer important insights into the identification of patient-specific seizure propagation pathways for pre-surgical evaluation, when a clear set of competing hypotheses exists. Further validation of this methodology could be achieved using more direct techniques such as intracranial recordings (Carmichael et al., 2010; Vulliemoz et al., 2011).

## Figures and Tables

**Table 1:** Posterior mean values of the estimated haemodynamic parameters obtained for the winning model in each ROI and BOLD sequence.

BOLD	ROI	Haemodynamic parameters^a^					
κ	γ	τ	α	E_0_	ϵ
Sequence 1	HH	0.671	0.413	1.024	0.323	0.341	− 0.026
PR	0.607	0.419	0.898	0.318	0.338	0.013
AR	0.636	0.417	0.890	0.317	0.337	0.026
Sequence 2	HH	0.651	0.415	0.977	0.321	0.340	− 0.011
PR	0.626	0.426	0.868	0.318	0.337	0.013
AR	0.612	0.424	0.849	0.317	0.336	0.026

a κ — rate constant of the vasodilatory signal decay, γ — rate constant for auto-regulatory feedback by blood flow, τ — transit time, α — Grubb's vessel stiffness exponent, E_0_ — capillary resting net oxygen extraction, ϵ — ratio of intra-extravascular BOLD signal.
